# Hematologic disorder: A manifestation of helicobacter pylori infection

**DOI:** 10.22088/cjim.8.2.133

**Published:** 2017

**Authors:** Mohammad Zamani, Jila Masrour- Roudsari, Vahid Zamani

**Affiliations:** 1 Student Research Committee, Babol University of Medical Sciences, Babol, Iran.; 2 Cancer Research Center, Babol University of Medical Sciences, Babol, Iran.; 3 Infectious Diseases and Tropical Medicine Research Center, Health Research Institute, Babol University of Medical Sciences, Babol, Iran.; 4 Vice-chancellery for Health, Babol University of Medical Sciences, Babol, Iran.

**Keywords:** Hematologic disorder, H.pylori, Gastric diseases

**Dear Editor,**

Colonization of *H.pylori* as a bacterial pathogen in the stomach can cause a number of gastric diseases, such as peptic ulcers, chronic gastritis and gastric cancer (1, 2). Over the past years, a series of systemic diseases has been reported to be potentially related to *H. pylori* infection, including cardiovascular, neurologic, hepatobiliary, dermatologic, endocrine and metabolic, immunologic, respiratory and urogenital diseases (3-6).

Hematologic disorders were other extragastric manifestations of *H. pylori*. Several studies presented that *H. pylori* can be a causative factor for anemia, iron deficiency and iron deficiency anemia (IDA) (3, 7, 8). In addition, it was stated that eradication of this pathogen not only led to increase in response to oral iron therapy and level of ferritin, but also could cure anemia completely in several cases with unexplained IDA (5, 8, 9). Idiopathic thrombocytopenic purpura (ITP) is the other hematologic disease shown to be related to infection of *H. pylori*. It was reported that treatment of the infection was associated with increased platelet count in some patients with ITP. Modulation of Fcγ-receptor balance of monocytes or macrophages, and mimicry of platelet antigens by *H. pylori* peptides are putative mechanisms explaining the relation between ITP and this infection (9). It was also proposed that *H. pylori-*induced gastritis could be a leading factor of vitamin B12 deficiency in patients (10). Altogether, ITP is considered as an accepted hematologic manifestation of *H. pylori* infection. Besides, it seems that the infection can possibly decrease level of iron storage, resulting in IDA. Therefore, *H. pylori* treatment can be indicated in patients with IDA, ITP and vitamin B12 deficiency.

## Conflict of interest

None declared.
